# Supplementary material for: Matching sensor ontologies through siamese neural networks without using reference alignment
Source: PeerJ Comput Sci. 2021 Jun 18;7:e602. doi: 10.7717/peerj-cs.602 (PMC8237319; doi:10.7717/peerj-cs.602)
Supplement: Supplemental Information 1 [file peerj-cs-07-602-s001.zip › 302/onto.html]

eBiquity Publication Ontology


# eBiquity Publication Ontology

Author:   
Contributor:   
Date:   
Version: 0.1

## Classes

**Resource** (Resource, *)*
:   - #title [1 1]
    - #publishedOn [0 1]
    - #description [0 1]
    - #version [0 1]
    - #author [1 +oo]
    - #firstAuthor [0 1]

    **Publication** (Publication, *)*
    :   - #editor [1 +oo]
        - #abstract [0 1]

**SoftCopy** (Soft Copy, *)*
:   - #softCopyFormat [1 1]
    - #softCopyURI [1 1]
    - #softCopySize [1 1]

**Article** (Article, *)*
:   super: *http://ebiquity.umbc.edu/v2.1/ontology/publication.owl#Publication*

**Book** (Book, *)*
:   super: *http://ebiquity.umbc.edu/v2.1/ontology/publication.owl#Publication*

**InBook** (InBook, *)*
:   super: *http://ebiquity.umbc.edu/v2.1/ontology/publication.owl#Publication*

**InCollection** (InCollection, *)*
:   super: *http://ebiquity.umbc.edu/v2.1/ontology/publication.owl#Publication*

**InProceedings** (InProceedings, *)*
:   super: *http://ebiquity.umbc.edu/v2.1/ontology/publication.owl#Publication*

**MastersThesis** (MastersThesis, *)*
:   super: *http://ebiquity.umbc.edu/v2.1/ontology/publication.owl#Publication*

**Misc** (Misc, *)*
:   super: *http://ebiquity.umbc.edu/v2.1/ontology/publication.owl#Publication*

**PhdThesis** (PhdThesis, *)*
:   super: *http://ebiquity.umbc.edu/v2.1/ontology/publication.owl#Publication*

**Proceedings** (Proceedings, *)*
:   super: *http://ebiquity.umbc.edu/v2.1/ontology/publication.owl#Publication*

**TechReport** (TechReport, *)*
:   super: *http://ebiquity.umbc.edu/v2.1/ontology/publication.owl#Publication*

## Properties

**author**: #Resource -> http://ebiquity.umbc.edu/v2.1/ontology/person.owl#Person *()*


**firstAuthor**: #Resource -> http://ebiquity.umbc.edu/v2.1/ontology/person.owl#Person *()*


**softCopy**: #Resource -> #SoftCopy *()*


**relatedProject**: #Resource -> http://ebiquity.umbc.edu/v2.1/ontology/project.owl#Project *()*


**editor**: #Publication -> http://ebiquity.umbc.edu/v2.1/ontology/person.owl#Person *()*

**title** #Resource -> http://www.w3.org/2001/XMLSchema#string *()*


**publishedOn** #Resource -> http://www.w3.org/2001/XMLSchema#dateTime *()*


**description** #Resource -> http://www.w3.org/2001/XMLSchema#string *()*


**keyword** #Resource -> http://www.w3.org/2001/XMLSchema#string *()*


**version** #Resource -> http://www.w3.org/2001/XMLSchema#string *()*


**type** #Resource -> http://www.w3.org/2001/XMLSchema#string *()*


**softCopyFormat** #Resource -> http://www.w3.org/2001/XMLSchema#string *()*


**softCopyURI** #Resource -> http://www.w3.org/2001/XMLSchema#anyURI *()*


**softCopySize** #Resource -> http://www.w3.org/2001/XMLSchema#nonNegativeInteger *()*


**abstract** #Publication -> http://www.w3.org/2001/XMLSchema#string *()*


**edition** #Publication -> http://www.w3.org/2001/XMLSchema#string *()*


**chapter** #Publication -> http://www.w3.org/2001/XMLSchema#string *()*


**series** #Publication -> http://www.w3.org/2001/XMLSchema#string *()*


**pages** #Publication -> http://www.w3.org/2001/XMLSchema#string *()*


**volume** #Publication -> http://www.w3.org/2001/XMLSchema#string *()*


**number** #Publication -> http://www.w3.org/2001/XMLSchema#string *()*


**note** #Publication -> http://www.w3.org/2001/XMLSchema#string *()*


**address** #Publication -> http://www.w3.org/2001/XMLSchema#string *()*


**organization** #Publication -> http://www.w3.org/2001/XMLSchema#string *()*


**journal** #Publication -> http://www.w3.org/2001/XMLSchema#string *()*


**booktitle** #Publication -> http://www.w3.org/2001/XMLSchema#string *()*


**school** #Publication -> http://www.w3.org/2001/XMLSchema#string *()*


**institution** #Publication -> http://www.w3.org/2001/XMLSchema#string *()*


**publisher** #Publication -> http://www.w3.org/2001/XMLSchema#string *()*


**counter** #Publication -> http://www.w3.org/2001/XMLSchema#nonNegativeInteger *()*

## Individuals

---

Generated by OWL2HTML
